# Antidepression and Prokinetic Effects of Paeoniflorin on Rats in the Forced Swimming Test via Polypharmacology

**DOI:** 10.1155/2020/2153571

**Published:** 2020-07-11

**Authors:** Dao-zhou Mu, Mei Xue, Jian-jun Xu, Ying Hu, Yi Chen, Ping Ren, Xi Huang

**Affiliations:** ^1^Jining No. 1 People's Hospital, Jining 272011, Shandong, China; ^2^Institute of TCM-related Comorbid Depression, Nanjing University of Traditional Chinese Medicine, Nanjing 210023, China; ^3^Hospital of Traditional Chinese Medicine of Zhongshan, Zhongshan 528400, China; ^4^Medical College of Xiamen University, Xiamen 361005, China; ^5^Jiangsu Province Hospital of Traditional Chinese Medicine, Nanjing 210029, China

## Abstract

Paeoniflorin, an organic compound extracted from the roots of the white peony (*Paeonia lactiflora*) plant, has previously been shown to exert antidepression and prokinetic effects. The traditional Chinese prescription Si-Ni-San, of which paeoniflorin is a constituent, is often used in treating depression and functional gastrointestinal disorders. The effectiveness of Si-Ni-San has been shown to be less effective in a paeoniflorin-deleted form. The present study further investigates whether paeoniflorin alone is as effective as herbal prescriptions in which the compound is a constituent, specifically any antidepressive and prokinetic effect on rats subjected to a forced swimming test (FST). The FST was used to establish the depression model. Sprague-Dawley rats were administrated with 10 mg/kg paeoniflorin by gastrogavage three times before the behavioral test and gastrointestinal motility tests, respectively. In antidepression studies, fluoxetine was used as the positive control. In order to determine the effect of paeoniflorin on the gastrointestinal movement, mosapride was used as the positive control. Plasma and hippocampus monoamine, hypothalamic-pituitary-adrenal axis, plasma brain-derived neurotrophic factor (BDNF), superoxide dismutase (SOD), methane dicarboxylic aldehyde (MDA), ghrelin, motilin, and hippocampus nitric oxide (NO) were assessed using an enzyme-linked immunosorbent assay (ELISA). Gastrointestinal (GI) motility was measured *in vivo* and *in vitro*. Rats subjected to FST showed decreased gastric emptying and intestinal transit *in vivo*, decreased plasma and hippocampus 5-hydroxytryptamine, norepinephrine, dopamine, ghrelin, motilin, and reduced plasma BDNF and SOD as well as increased plasma and hippocampus corticotropin-releasing hormone, adrenocorticotropic hormone, corticosterone, plasma MDA, and hippocampus NO. Paeoniflorin reversed these symptoms in a similar manner to fluoxetine and mosapride, respectively. *In vitro*, paeoniflorin can stimulate the jejunal contract of healthy rats dose-dependently. The results suggest that paeoniflorin can simultaneously exert antidepression and prokinetic effects via polypharmacology.

## 1. Introduction

The traditional Chinese prescription Si-Ni-San is often used in treating depression [1] and functional gastrointestinal disorders (FGIDs) [2]. Chaihu-Shugan-San (CSS), which is derived from Si-Ni-San, can simultaneously exert antidepression and prokinetic effects [3]. These multitarget effects of Chinese formulae may provide a more effective alternative to Western medicine in treating comorbid depression and FGIDs. Paeoniflorin is the most effective component of the two formulae. Research has shown that the paeoniflorin-deleted Si-Ni-San was significantly less effective than Si-Ni-San and paeoniflorin while paeoniflorin exhibited a similar effect to Si-Ni-San [4]. We were, therefore, very interested to test whether paeoniflorin can induce antidepression and prokinetic effects similar to Si-Ni-San and CSS. A previous study has reported that paeoniflorin can modulate the hypothalamic-pituitary-adrenal (HPA) axis and upregulate the serotonergic and noradrenergic systems of rats exposed to chronic unpredictable stress, which may elucidate the mechanisms of the antidepressive effect of paeoniflorin [5]. Paeoniflorin can also reduce the immobility time in forced swimming and tail suspension tests of mice and antagonize reserpine-induced ptosis, akinesia, and hypothermia, while upregulating serotonergic systems [6]. In the menopause depression model of rats, paeoniflorin can exert antidepressive effects through upregulating 5-HT1AR expression, while downregulating 5-HT2AR expression in the brains of rats [7]. There is, however, no report of both antidepressive and prokinetic effects of paeoniflorin on rats subjected to a forced swimming test (FST). Thus, in the present study, we used a 15 min FST to induce the depression-like behavioral response of rats and investigate the antidepressive and prokinetic effect of paeoniflorin on rats subjected to FST. Monoamine neurotransmitter and HPA axis were measured, while plasma brain-derived neurotrophic factor (BDNF), superoxide dismutase (SOD), methane dicarboxylic aldehyde (MDA), and hippocampus nitric oxide (NO) were detected to investigate the mechanism of the antidepressive effect of paeoniflorin. In addition, plasma ghrelin and motilin were assessed to elucidate the mechanism of the prokinetic effect of paeoniflorin.

## 2. Materials and Methods

### 2.1. Animals

Healthy adult male Sprague-Dawley (SD) rats weighing 180–210 g (about 7 weeks old, SPF grade, offered by Shanghai Jie-si-jie Experimental Animals Co. Limited) were caged to acclimatize to their new environment for 7 days prior to experiments. All the experiments were conducted per the relevant ethical guidelines of the Experimental Animal Ethics Committee of Nanjing University of Traditional Chinese Medicine (permit number: ACU-42 (20141229)). A 12-hour light/dark cycle was maintained (lights on: 7 : 00–19 : 00; lights off: 19 : 00–7:00). The humidity was 55 ± 2%, and the temperature was 21–25°C. All the rats were given free access to water and food prior to experiments. Following the drawing of experimental samples, rats were euthanized via intraperitoneal injection using a solution of 2% pentobarbital sodium 60 mg/kg weight. A total of 101 rats were used in the experiments. The experiments were carried out from September 22, 2015, to March 9, 2016. The animals' health and behavior were monitored daily.

### 2.2. Drug

Paeoniflorin (purity 98%) was purchased from Nanjing Jingzhu Bio-science and Technology Co. Limited (Jiangsu, China). Fluoxetine (as a positive control in antidepression tests) [7] and mosapride (as a positive control in prokinetic tests) [8] were bought from the Jiangsu Provincial Hospital of Traditional Chinese Medicine. All three drugs were prepared in ultrapure water at concentrations of 1 mg/ml, 2 mg/ml, and 1 mg/ml, respectively. Although fluoxetine and mosapride are not completely soluble in water, they disintegrate, forming a suspension. Before use, the suspension was shaken to fully suspend the powder in the water.

### 2.3. Forced Swimming Test (FST)

The FST was carried out following the method of Porsolt et al. [9]. Rats were forced to swim 15 min individually in a cylinder (50 cm × 25 cm) which was filled with water at a temperature of 25°C to a depth of 30 cm. The cylinder was cleaned after each test. After 24 h, the Depression Scan RH type animal despair automatic analyzer system (US, Clever) was used to record the immobility time of rats during a 5 min FST. Immobility time refers to the time during which rats stopped struggling and floated motionlessly or only tried to put their heads above water.

### 2.4. Open-Field Test (OFT)

The OFT was performed in an open field apparatus (50 cm × 50 cm × 50 cm). The base was divided into a central area and a peripheral area by computer. Rats were placed in the center of the apparatus, one rat per open field. The TopScanHR type animal track and behavior intelligent analysis system was used to record the moving distance during 5 min in the apparatus. Ethyl alcohol was used to clean the apparatus after each test.

### 2.5. Antidepression Experiment

Forty SD rats were divided randomly into four groups of ten, including a normal control (NC) group, a model (M) group, a fluoxetine (F) group, and a paeoniflorin (P) group. Apart from the NC group, rats in other groups were subjected to a single 15 min FST. Normal saline (1 ml/100 g), fluoxetine (20 mg/kg), and paeoniflorin (10 mg/kg) were orally administered to rats in the M group, F group, and P group, respectively, at three times: 24 h, 5 h, and 1 h before the behavioral test. After 24 h, OFT and 5 min forced swimming immobility time were used to evaluate the depression-like behavior of rats. All rats were deprived of food and fed with 5% glucose water only for 24 h before the behavioral test. Rats were sacrificed 30 min after the behavioral test. Blood samples were drawn from the abdominal aorta and placed in tubes with heparin which were then centrifuged at 3000 rpm for 10 min at 4°C. Supernatants were collected and stored at −80°C until assayed. Plasma monoamines and corticotropin-releasing hormone (CRH), adrenocorticotropic hormone (ACTH), corticosterone (CORT), BDNF, SOD, and MDA were assessed using enzyme-linked immunosorbent assay (ELISA). Simultaneously, rats were decollated and their hippocampi were carefully removed in order to detect monoamines, CRH, ACTH, CORT, and NO in the hippocampus using ELISA. The hippocampi were stored at −80°C prior to testing.

### 2.6. Measurement of GI Movement *In Vivo*

This test was carried out according to previous research [10, 11]. Rats, 51 in total, were divided randomly into five groups: NC group, M group, F group, P group, and mosapride group (Mo group). (The NC group contained 11 rats while each of the other groups contained 10 rats.) All rats, except those in the NC group, were subjected to a 15 min FST. Normal saline (1 ml/100 g), fluoxetine (20 mg/kg), paeoniflorin (10 mg/kg), and mosapride (10 mg/kg) were given by gavage at 24 h, 5 h, and 0.5 h prior to the gastrointestinal motility test. All rats fasted and were fed only 5% glucose water 24 h prior to the GI motility test. 30 min after the last administration, 1 ml semiliquid nonnutrient dye (Evans blue 50 mg/ml dissolved in 0.5% methylcellulose) was orally administered to all rats. Blood samples were collected from orbit. 20 min later, all rats were anesthetized via intraperitoneal injection using a solution of 2% pentobarbital sodium 60 mg/kg weight. Gastric emptying (GE) and intestinal transit (IT) were measured per a previous report [12, 13]. Plasma motilin was assayed to investigate the mechanism of paeoniflorin's effect on the gastrointestinal (GI) motility of rats in the FST.

### 2.7. Measurement of GI Movement *In Vitro*

To further determine the effect of paeoniflorin on GI movement in rats, we observed the contractile activity of rat jejunum *in vitro*. A section of the jejunum was removed from healthy SD rats and cut into 2 cm segments. The segments were immediately placed into Krebs-bicarbonate buffer (4.8 mM KCl, 118 mM NaCl, 1.2 mM MgSO_4_, 1.2 mM KH_2_PO_4_, 2.5 mM CaCl_2_, 11 mM glucose, and 25 mM NaHCO_3_, pH 7.4, at 37°C) while being oxygenated continuously (5% CO_2_, 95% O_2_). The segments of jejunum were then rapidly fixed in organ bath chambers which were filled with oxygenated Krebs-bicarbonate buffer, one segment per chamber. The lower end was fixed at the bottom of the chamber and the upper end was linked to a transducer. A multilead physiological recording instrument (AD Instruments, Australia) was used to measure the mobility of the jejunum segments. Varying doses of paeoniflorin were added to the organ bath chamber in increasing concentration (0.5 mg/ml, 1 mg/ml, 2 mg/ml, and 4 mg/ml), without washing between each administration. Fluoxetine (2 mg/ml) was added alone to another chamber.

### 2.8. Statistical Analysis

SPSS 16.0 was used. Data were shown as mean ± standard deviation ($\bar{\chi }$ ± SD) and one-way analysis of variance (ANOVA) was used to evaluate the data. Individual comparisons were then made using the post hoc SNK and LSD tests where appropriate. *p* < 0.05 indicated a statistically significant difference.

## 3. Results

### 3.1. Behavioral Response

#### 3.1.1. Immobility Time in 5 min Forced Swimming Test

**Figure 1:**
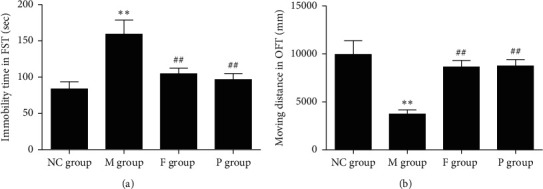
Effect of paeoniflorin on immobility time in FST and moving distance in OFT of rats with FST. (a) Immobility time of the M group significantly increased compared with the NC group, while that of the P group and F group markedly decreased compared with the M group. (b) Moving distance in OFT significantly decreased in M group compared with the NC group, while P and F can reverse this change. Compared with the NC group, ^*∗∗*^*p* < 0.01. Compared with the M group, ^##^*p* < 0.01. NC, normal control; M, model; P, paeoniflorin; F, fluoxetine; OFT, open-field test; FST, forced swimming test.

Compared with the NC group, rats in the M group demonstrated a significant increase in immobility following 5 min FST (M group: 159.14 ± 19.66 vs NC group: 83.68 ± 9.91, *p* < 0.01). Fluoxetine and paeoniflorin significantly reduced the immobility time compared with the M group (F group: 104.80 ± 7.62 vs M group: 159.14 ± 19.66, *p* < 0.01; P group: 96.71 ± 8.27 vs M group: 159.14 ± 19.66, *p* < 0.01). These data are shown in Figure 1.

#### 3.1.2. Open-Field Test (OFT)

Compared with the NC group, rats in the M group exhibited significantly decreased mobility in the OFT (M group: 3719.31 ± 451.27 vs NC group: 9933.45 ± 1432.08, *p* < 0.01). Fluoxetine and paeoniflorin increased the moving distance remarkably compared with the M group (F group: 8628.42 ± 682.73 vs M group: 3719.31 ± 451.27, *p* < 0.01; P group: 8732.10 ± 655.64 vs M group: 3719.31 ± 451.27, *p* < 0.01) (Figure 1).

### 3.2. Gastrointestinal (GI) Movement

#### 3.2.1. *In Vivo* Effects of Paeoniflorin on GI Movement of Rats Undergoing FST

Compared with rats in the NC group, rats in the M group showed significantly decreased GE and IT (GE: NC group 0.6451 ± 0.0558 vs M group 0.4870 ± 0.0323, *p* < 0.01; IT: NC group 0.695 ± 0.137 vs M group 0.371 ± 0.037, *p* < 0.01).

**Figure 2:**
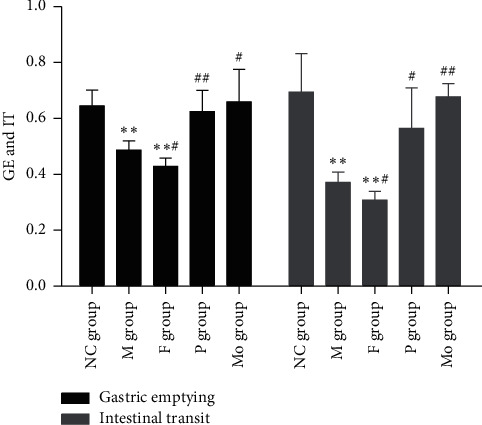
Effects of paeoniflorin on GE and IT on rats subjected to FST. GE and IT of the M group and F group significantly decreased compared with the NC group, while P and Mo can obviously increase GE and IT compared with the M group. Compared with the NC group, ^*∗∗*^*p* < 0.01. Compared with the M group, ^##^*p* < 0.01 and ^#^*p* < 0.05. M, model; F, fluoxetine; NC, normal control; P, paeoniflorin; Mo, mosapride; FST, forced swimming test.

Paeoniflorin can significantly promote gastrointestinal motility (GE: P group 0.6247 ± 0.0754 vs M group 0.4870 ± 0.0323, *p* < 0.01; IT: P group 0.565 ± 0.145 vs M group 0.371 ± 0.037, *p* < 0.05), in a manner similar to mosapride (GE: Mo group 0.6599 ± 0.1152 vs M group 0.4870 ± 0.0323, *p* < 0.05; IT: Mo group 0.677 ± 0.048 vs M group 0.371 ± 0.037, *p* < 0.01). Fluoxetine, however, further delayed GI movement compared with rats in the M group (GE: F group 0.4288 ± 0.0290 vs M group 0.4870 ± 0.0323, *p* < 0.05; IT: F group 0.308 ± 0.031 vs M group 0.371 ± 0.037, *p* < 0.05). These data are shown in Figure 2.

#### 3.2.2. *In Vitro* Effects of Paeoniflorin, Fluoxetine, and Neostigmine on Jejunal Smooth Muscle from Healthy Rats

**Figure 3:**
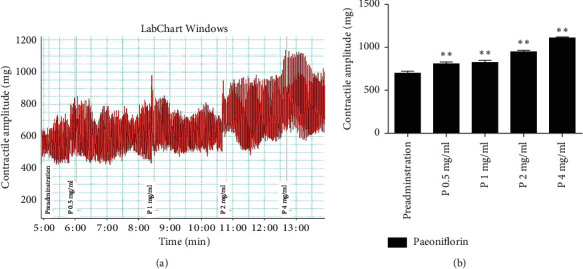
(a) Effects of paeoniflorin on the contractile amplitude of jejunal muscle from healthy rats *in vitro*. (b) Paeoniflorin can significantly increase the contractile amplitude of rat jejunal smooth muscle *in vitro*. P: paeoniflorin. ^*∗∗*^*p* < 0.01, compared with preadministration.

Compared with the preadministration of paeoniflorin, the contractile amplitude of rat jejunal smooth muscle significantly increased following the administration of paeoniflorin of varying doses, and this effect was dose-dependent: preadministration of paeoniflorin: 697.6753 ± 24.9537 vs paeoniflorin 0.5 mg/ml: 808.1867 ± 20.7491 vs paeoniflorin 1 mg/ml: 823.7092 ± 24.4624 vs paeoniflorin 2 mg/ml: 946.8912 ± 18.4724 vs paeoniflorin 4 mg/ml: 1108.7980 ± 12.0524 (Figures 3(a) and 3(b)). The higher the concentration of paeoniflorin administered, the stronger the resulting contraction.

**Figure 4:**
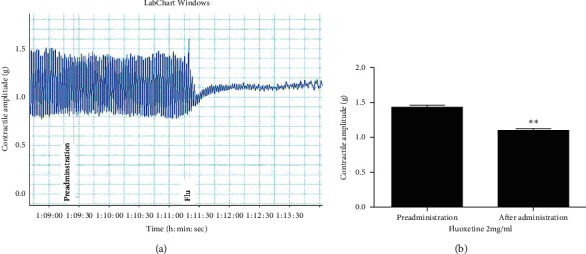
(a) Effects of fluoxetine on the contractile amplitude of jejunal muscle from healthy rats *in vitro*. (b) Fluoxetine can noticeably decrease the contractile amplitude of jejunal muscle *in vitro*. Flu: fluoxetine. ^*∗∗*^*p* < 0.01, compared with preadministration.

**Figure 5:**
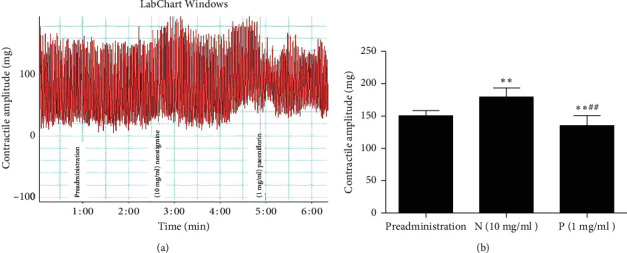
(a) Effects of paeoniflorin and neostigmine on the contractile amplitude of jejunal muscle from healthy rats *in vitro*. (b) Neostigmine can increase the contractile amplitude of jejunal muscle *in vitro*, but this change can be reversed by paeoniflorin. N: neostigmine; P: paeoniflorin. ^*∗∗*^*p* < 0.01, compared with preadministration; ^*##*^*p* < 0.01, compared with N.

Conversely, fluoxetine significantly reduced the contractile amplitude of rat jejunal muscle: preadministration: 1.4326 ± 0.0275 compared with after administration: 1.1001 ± 0.0257 (Figures 4(a) and 4(b)). When neostigmine was used to stimulate jejunal muscle contraction prior to paeoniflorin, however, the effect of neostigmine was inhibited: preadministration: 150.59 ± 8.003 vs neostigmine: 179.78 ± 13.652 vs paeoniflorin: 135.35 ± 15.430 (Figures 5(a) and 5(b)).

### 3.3. Effect of Paeoniflorin on Monoamines, HPA Axis, Plasma BDNF, and Hippocampus NO

#### 3.3.1. Effect of Paeoniflorin on Monoamines in Plasma and Hippocampus

**Figure 6:**
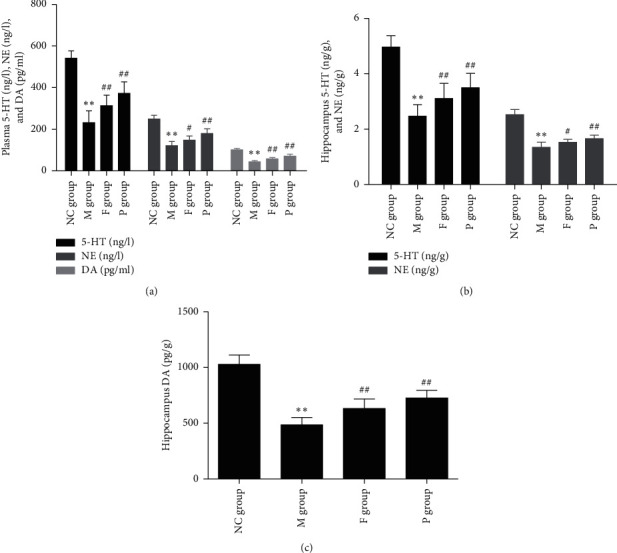
Effects of paeoniflorin on plasma and hippocampus monoamines of rats subjected to FST. (a) Plasma 5-HT, NE, and DA remarkably reduced in the M group compared with the NC group, while F and P can reverse these changes. (b) *Hippocampus* 5-HT and NE obviously decreased in the M group, while F and P can reverse these changes. (c) *Hippocampus* DA significantly decreased in the M group, while F and P can reverse this change. Compared with the NC group, ^*∗∗*^*p* < 0.01. Compared with the M group, ^##^*p* < 0.01 and ^#^*p* < 0.05. NC, normal control; M, model; F, fluoxetine; P, paeoniflorin; 5-HT, 5-hydroxytryptamine; NE, norepinephrine; DA, dopamine; FST, forced swimming test.

Compared to the control group, 5-hydroxytryptamine (5-HT), norepinephrine (NE), and dopamine (DA) in rats with FST decreased in both plasma and hippocampus (*p* < 0.01). Compared with the model group, paeoniflorin and fluoxetine can reverse these symptoms: fluoxetine group, plasma NE, and hippocampus NE, *p* < 0.05; others all *p* < 0.01. In this respect, the effect of paeoniflorin is superior to fluoxetine. These data are shown in Figure 6.

#### 3.3.2. Effects of Paeoniflorin on HPA Axis

**Figure 7:**
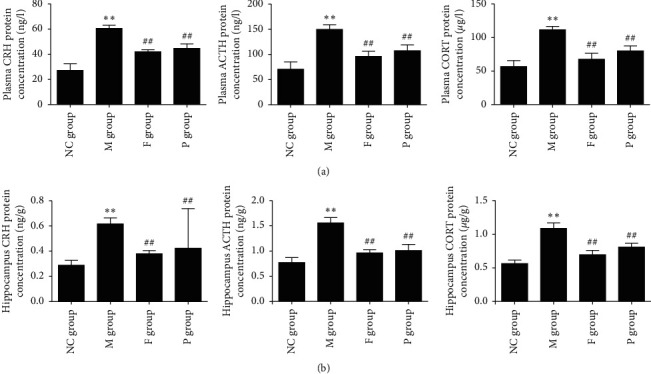
Effects of paeoniflorin on the HPA axis of rats subjected to FST. (a) Plasma CRH, ACTH, and CORT significantly increased in M group, while F and P can reverse these changes. (b) Hippocampus CRH, ACTH, and CORT increased markedly in the M group, while F and P can reverse these changes. Compared with the NC group, ^*∗∗*^*p* < 0.01. Compared with the M group, ^##^*p* < 0.01; NC, normal control; M, model; F, fluoxetine; P, paeoniflorin; HPA, hypothalamic-pituitary-adrenal; FST, forced swimming test; CRH, corticotropin-releasing hormone; ACTH, adrenocorticotropic hormone; CORT, corticosterone.

Compared with the NC group, the HPA axis of rats in the M group showed higher activity. CRH, ACTH, and CORT were significantly increased in both the plasma and hippocampus of rats in the M group (*p* < 0.01). Paeoniflorin and fluoxetine can reverse these symptoms. In this respect, the effect of fluoxetine is superior to paeoniflorin. These data are shown in Figure 7.

#### 3.3.3. Effect of Paeoniflorin on Plasma BDNF of Rats with FST

**Figure 8:**
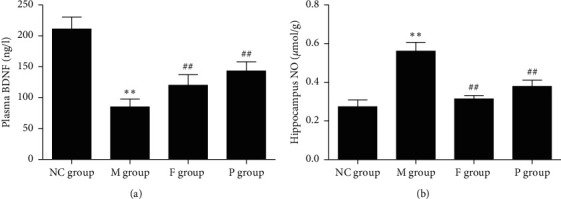
Effects of paeoniflorin on plasma BDNF and hippocampus NO of rats subjected to FST. (a) Plasma BDNF was significantly downregulated in the M group, while F and P can reverse this change. (b) Hippocampus NO obviously increased in the M group, while F and P can reverse this change. Compared with the NC group, ^*∗∗*^*p* < 0.01. Compared with the M group, ^##^*p* < 0.01. BDNF, brain-derived neurotrophic factor; NO, nitric oxide; FST, forced swimming test; M, model; F, fluoxetine; P, paeoniflorin.

Compared with the NC group, rats in the M group showed decreased plasma BDNF (*p* < 0.01). Compared with the M group, paeoniflorin and fluoxetine can increase plasma BDNF of rats with FST (*p* < 0.01). In this respect, the effect of paeoniflorin is superior to that of fluoxetine (*p* < 0.01). These data are shown in Figure 8.

#### 3.3.4. Effects of Paeoniflorin on Hippocampus NO of Rats with FST

Compared with the NC group, hippocampus NO increased significantly in rats in the M group (*p* < 0.01). Paeoniflorin and fluoxetine can decrease the hippocampus NO of rats with FST (*p* < 0.01). In this respect, the effect of paeoniflorin is inferior to that of fluoxetine (*p* < 0.01). These data are shown in Figure 8.

#### 3.3.5. Effects of Paeoniflorin on Plasma SOD and MDA

Compared with the NC group, rats in the M group showed increased plasma MDA and decreased plasma SOD (*p* < 0.01). Compared with the M group, paeoniflorin can elevate plasma SOD, which is superior to fluoxetine (*p* < 0.01), and reduce plasma MDA, which is inferior to fluoxetine (*p* < 0.01) (Table 1).

#### 3.3.6. Effects of Paeoniflorin on Plasma Motilin of Rats with FST

**Figure 9:**
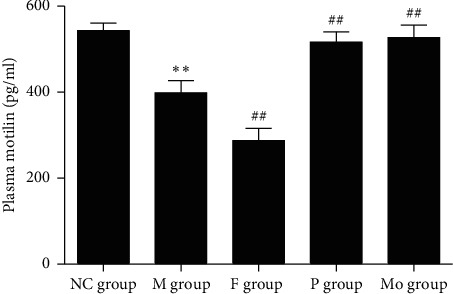
Effects of paeoniflorin on plasma motilin of rats subjected to FST. Plasma motilin significantly decreased in M group compared with the NC group and paeoniflorin can reverse this change. Conversely, fluoxetine further decreased plasma motilin. Compared with the NC group, ^*∗∗*^*p* < 0.01. Compared with the M group, ^##^*p* < 0.01. FST, forced swimming test; M, model; NC, normal control.

Compared with the NC group, forced swimming stress significantly decreased plasma motilin (*p* < 0.01). Compared with the M group, fluoxetine further reduced plasma motilin of rats subjected to FST (*p* < 0.01). Paeoniflorin and mosapride can increase plasma motilin of rats with FST (*p* < 0.01), a result not significantly different from the NC group (Figure 9).

## 4. Discussion

The results of the experiments in this study indicate that paeoniflorin can simultaneously exert antidepressant-like and prokinetic effects via polypharmacology.

We found that rats undergoing 15 min forced swimming stress showed increased immobility time following a subsequent 5 min FST and decreased mobility in the OFT. Paeoniflorin and fluoxetine can reverse these symptoms. These behavioral changes suggest that the FST can successfully induce a depression-like behavioral response of rats and evaluate the antidepressive effect of drugs. These findings are consistent with previous reports by members of our research team [3, 8].

We also found that paeoniflorin exerted antidepressive and prokinetic effects simultaneously on rats with or without FST. The antidepressive effect of paeoniflorin was similar to fluoxetine and its prokinetic effect was identical to that of mosapride. These are significant results for new drugs in treating depression, especially comorbid depression and functional gastrointestinal disorder. Compared with Western medicine, which acts on single targets, traditional Chinese prescriptions act on multitargets. This suggests that Chinese medicine may provide alternative treatments to Western medicine in complicated clinical cases. Previous reports and these results provide evidence that monosome drugs extracted from natural herbs can also have multieffects similar to their maternal formula while also having little in the way of side effects.

Low concentrations of monoamines in both plasma and hippocampus are often used to explain the pathology of depression. Antidepressants such as SSRI and MAOI can exert an antidepressive effect through the upregulation of monoamines. Qiu et al. [5] reported that paeoniflorin can exert antidepressant-like effects through modulation of the HPA axis and upregulation of serotonergic and noradrenergic systems on rats exposed to chronic unpredictable stress. In this study, we provide evidence that stress can induce depression-like behavior and decrease plasma and hippocampus monoamines. Paeoniflorin was found to upregulate plasma and hippocampus monoamines and this effect was better than using fluoxetine.

Hyperactivity of the HPA axis is another cause of depression. Previous studies indicate that paeoniflorin can significantly improve the depression-like behavior of rats with menopausal depression through downregulating the activity of the HPA axis [7]. This research demonstrated that paeoniflorin could decrease the concentration of CRH, ACTH, and CORT in both plasma and hippocampus in rats undergoing forced swimming stress, which was similar to the effect of fluoxetine. The effect of paeoniflorin was, however, inferior to the effect of fluoxetine.

BDNF is expressed extensively in the cerebral cortex and hippocampus and is important for neuronal proliferation, differentiation, nutrition, and maturity. Serum BDNF levels decreased in patients with major depression and increased after symptoms were relieved due to treatment with antidepressants [14–16]. This research demonstrated that plasma BDNF decreased in rats subjected to 15 min forced swimming stress and increased after being treated with paeoniflorin and fluoxetine, as well as relieving depression-like behavior in rats. The effect of paeoniflorin was superior to fluoxetine.

In physiological conditions, as a neurological messenger molecule, NO plays a role in regulating physiological functions. In pathological conditions, however, too much NO can cause or worsen a neuron's damage; even death of the cell may result. Plasma NO was increased in male patients with major depression and male rats exposed to chronic unpredicted mild stress [17] and significantly decreased after being treated with antidepressants [18]. Bacillus Calmette-Guerin (BCG) can induce depression in mice, while Ibuprofen can attenuate the depressive-like behavior of mice by decreasing the cerebral NO levels [19]. This research demonstrated that the NO concentration in the hippocampus was clearly increased after stress, a result consistent with previous reports. Paeoniflorin and fluoxetine can reverse this change, which may elucidate the mechanism of the antidepressant-like effect of paeoniflorin. In this respect, paeoniflorin was inferior to fluoxetine.

Under normal conditions, the human body may produce free radicals, and these free radicals will be cleared by the antioxidative system. Too high a level of free radicals will be produced when the human body is exposed to various types of stress, resulting in oxidative damage. SOD can eliminate free radicals, while MDA is an indicator of too high a level of free radicals. Oxidative stress refers to the imbalance between the production of free radicals and the antioxidant system. Meta-analysis has shown that oxidative stress plays an important role in the pathology of major depressive disorders and the effect of antidepressants may be related to the improvement of oxidative stress state or the antioxidant effect [20]. The traditional Chinese medicine formulation CSS is often used clinically to treat depression and functional gastrointestinal disorder, and its antidepressive effect may be related to its antioxidant activity [21]. In this study, we found that plasma SOD decreased, while plasma MDA increased in rats of the M group. Paeoniflorin and fluoxetine were able to reverse this change, suggesting that the antidepressive effect of paeoniflorin may be related to its upregulating of SOD and downregulating of MDA.

Motilin, named because of its stimulation of gastric motility, is a polypeptide hormone secreted by M cells in the proximal small intestine. It is a 22-amino acid protein. Motilin plays a role in the phase III stage of the migrating motor complex (MMC). Phase III may enable the stomach and intestine to eliminate the undigested content and can prevent bacteria from overgrowing in the proximal intestine. It may be related to the sense of hunger [22]. Low concentrations of motilin can stimulate the function of neurons in the upper intestine, while higher levels of motilin can directly promote the gut muscle's contraction [23]. In this study, we found that plasma motilin decreased in rats with FST, and paeoniflorin can reverse this change. Conversely, fluoxetine further decreased plasma motilin. These phenomena may explain the mechanism of the prokinetic effect of paeoniflorin.

The experiment which measured the effect of paeoniflorin on GI motility *in vitro* demonstrated that paeoniflorin can directly contract the jejunal muscle dose-dependently. This method excluded the factors in the internal environment of the body such as drug metabolism and other endocrine factors which may affect the results. We were, therefore, able to observe the direct action on jejunal muscles of paeoniflorin at varying doses.

In summary, paeoniflorin can attenuate the depression-like behavior of rats subjected to 15 min forced swimming stress (Figure 1). This may be due to its upregulation of plasma and hippocampus monoamines (Figure 6), plasma BDNF (Figure 8), and SOD (Table 1) as well as its downregulation of plasma MDA (Table 1), hippocampus NO (Figure 8), and activity of HPA axis (Figure 7). The antidepressive effects of paeoniflorin, as reported by Huang et al., may be due to its downregulation of HPA [7]. Moreover, paeoniflorin can promote GI motility both *in vivo* and *in vitro*, which may be related to its upregulation of plasma motilin (Figures 2–5 and 9). Previous findings have shown that paeoniflorin was the main constituent of Bai-Shao, which played an indispensable role in the antidepressant effect of CSS [24]. Previous studies have also demonstrated that CSS may cause antidepressive effects via regulation of the hippocampal BDNF-TrkB-ERK/Akt signaling pathway [25], while our research has shown that paeoniflorin can exert antidepressive and prokinetic effects similar to those found by CSS [3].

In future studies, we aim to investigate the antidepression and prokinetic effects of paeoniflorin on rats exposed to chronic unpredicted stress.

## Figures and Tables

**Table 1:** Effects of paeoniflorin on plasma SOD and MDA of rats with FST ($\bar{\chi }$ ± SD).

Group	*n*	SOD (U/ml)	*n*	MDA (nmol/l)
NC group	9	104.50 ± 7.57	10	5.29 ± 0.75
M group	10	47.93 ± 8.98^*∗∗*^	10	11.11 ± 0.78^*∗∗*^
F group	9	63.21 ± 8.17^##^	9	6.38 ± 0.59^##^
P group	10	74.33 ± 7.67^##^	10	7.48 ± 0.75^##^
*p* value		0.000		0.000

Data are shown as mean ± standard deviation ($\bar{\chi }$ ± SD), and one-way analysis of variance (ANOVA) followed by a least significant difference (LSD) test was used to evaluate the data. *p* < 0.05 indicated a statistically significant difference. Compared with the NC group, ^*∗∗*^*p* < 0.01; compared with the M group, ^##^*p* < 0.01. SOD: superoxide dismutase; MDA: methane dicarboxylic aldehyde; FST: forced swimming test; NC: normal control; M: model; F: fluoxetine; P: paeoniflorin.

## Data Availability

The statistical data used to support the findings of this study are included within the article and are available from the corresponding author upon request.
